# Relevance and Regulation of Alternative Splicing in Plant Heat Stress Response: Current Understanding and Future Directions

**DOI:** 10.3389/fpls.2022.911277

**Published:** 2022-06-23

**Authors:** Remus R. E. Rosenkranz, Sarah Ullrich, Karin Löchli, Stefan Simm, Sotirios Fragkostefanakis

**Affiliations:** ^1^Molecular Cell Biology of Plants, Goethe University Frankfurt, Frankfurt, Germany; ^2^Institute of Bioinformatics, University Medicine Greifswald, Greifswald, Germany

**Keywords:** pre-mRNA, alternative splicing, high temperature, co-transcriptional regulation, gene expression, splicing regulation

## Abstract

Alternative splicing (AS) is a major mechanism for gene expression in eukaryotes, increasing proteome diversity but also regulating transcriptome abundance. High temperatures have a strong impact on the splicing profile of many genes and therefore AS is considered as an integral part of heat stress response. While many studies have established a detailed description of the diversity of the RNAome under heat stress in different plant species and stress regimes, little is known on the underlying mechanisms that control this temperature-sensitive process. AS is mainly regulated by the activity of splicing regulators. Changes in the abundance of these proteins through transcription and AS, post-translational modifications and interactions with exonic and intronic *cis*-elements and core elements of the spliceosomes modulate the outcome of pre-mRNA splicing. As a major part of pre-mRNAs are spliced co-transcriptionally, the chromatin environment along with the RNA polymerase II elongation play a major role in the regulation of pre-mRNA splicing under heat stress conditions. Despite its importance, our understanding on the regulation of heat stress sensitive AS in plants is scarce. In this review, we summarize the current status of knowledge on the regulation of AS in plants under heat stress conditions. We discuss possible implications of different pathways based on results from non-plant systems to provide a perspective for researchers who aim to elucidate the molecular basis of AS under high temperatures.

## The Basis of Heat Stress Response in Plants

Exposure of a plant to temperatures that exceed a threshold can cause heat stress (HS) which can negatively affect growth and alter developmental transitions such as flowering time (Wahid et al., 2007). HS is caused either by short acute exposure to high temperatures, typically >15°C above the optimum, or for a prolonged period to mild temperature increases (~10°C) (Yeh et al., 2012). The temperature regimes that cause HS and the intensity of the effects of HS are dependent on the species, developmental stage and even tissue or cell type (Bokszczanin and Fragkostefanakis, 2013).

The survival from heat stress is based on the ability of the plant to activate the “heat stress response” (HSR), a set of molecular pathways that aim to direct cellular metabolism primarily toward the synthesis of end products with protective functions for heat sensitive cellular structures and macromolecules (Parsell and Lindquist, 1993; Baniwal et al., 2004; Ohama et al., 2017). The massive changes in cellular activities are manifested by the effect of heat stress on transcription. Hundreds of genes are induced upon exposure to HS (Schramm et al., 2006; Larkindale and Vierling, 2007; Liu and Charng, 2013; Hu et al., 2020a). The vast numbers compared to other abiotic stress responses reflect the acute nature of HS and its severity, but also the re-adjustment of molecular activities that are required for thermotolerance. Several transcription factors belonging to different protein families are involved in the upregulation of these genes (Baniwal et al., 2004; Liu et al., 2011; Fragkostefanakis et al., 2015a; Ohama et al., 2015). Among them, heat stress transcription factors (HSF) are considered as indispensable as they control the expression of the majority of cellular heat shock proteins, the chaperone/guardians of protein homeostasis (Scharf et al., 2012). Other factors play important roles as well, e.g., by regulating sets of HSPs, such as bZIP28, bZIP60 and DREB2A to name a few (Howell, 2013; Ohama et al., 2017). Beyond HSPs, genes coding for proteins involved in post-translational modifications, hormone signaling, and various metabolic pathways have been reported to be induced by HS (Busch et al., 2005; Larkindale and Vierling, 2007; Fragkostefanakis et al., 2015b; Keller et al., 2018; Hu et al., 2020a). Therefore, many of these changes are mirrored both at protein and metabolite levels (Majoul et al., 2004; Keller et al., 2018; Fragkostefanakis et al., 2019; Serrano et al., 2019; Paupière et al., 2020).

Many temperate plants reach the maximum of transcriptional induction after 1 h at 38–40°C, which is therefore considered as the optimum time and temperature window to monitor HSR regarding transcription (Scharf and Nover, 1982). However, other cellular processes such as translation follow different dynamics (Hahn et al., 2011). For example, protein translation is by and large stalled during HS, and is re-initiated when plants return to physiological temperatures (Shalgi et al., 2013; Zhang et al., 2017).

The ability of plants to activate HSR and survive an acute stress treatment is called basal thermotolerance (BTT), and typically under laboratory conditions is determined by an approximately 1 h exposure of, e.g., young seedlings at 40–50°C (Hong and Vierling, 2000; Yeh et al., 2012; Mesihovic et al., 2016). The limits of BTT can be extended if plants are pre-acclimated to a non-lethal stress regime (e.g., 38°C/30 min) which allows the induction of HSR and the accumulation of important transcription factors and chaperones which support HSR under an otherwise lethal stress treatment. This type is called acquired thermotolerance (ATT) and it can last up to 2 days, the period that cells typically can maintain pre-synthesized HSFs and HSPs after the conclusion of the pre-acclimation step (Burke et al., 2000). ATT resembles the acclimation achieved by a gradual temperature increase in temperature as occurs in nature (Larkindale and Vierling, 2007). Recent studies shown that acclimated plants develop a type of somatic memory which can be maintained for a period of approximately 7–14 days (Lämke et al., 2016). This type of memory is dependent on changes in chromatin structure and associated with histone modifications on memory related loci (Brzezinka et al., 2016; Lämke and Bäurle, 2017; Liu et al., 2019).

## Constitutive and Alternative Pre-mRNA Splicing

Pre-mRNA splicing is a central mechanism for regulation of gene expression in eukaryotes. The process is carried out by the spliceosome, a large RNA-protein-complex consisting of five small nuclear RNAs (snRNAs: U1, U2, U4, U5, and U6) and hundreds of non-snRNP proteins (Reddy et al., 2013; Staiger and Brown, 2013). The early spliceosome assembles by recognition of the 5′ and 3′ splice sites by U1 and U2 snRNP, respectively, followed by assembly with the U4/U6/U5- tri-snRNP to form the full spliceosome (Wilkinson and Charenton, 2020). Four consensus core *cis*-elements are considered central for constitutive pre-mRNA splicing: 5′splice site (5′SS), 3′splice site (3′SS), the branch point (BP) and the polypyrimidine tract (PPT) (Lorković et al., 2000; Reddy, 2007). Compared to yeast and mammals, pre-mRNA splicing is less understood in plants. While the core process is considered to be similar, the presence of a higher number of orthologs of spliceosome components in plant genomes indicate differences as well (Wang and Brendel, 2004; Barta et al., 2010; Richardson et al., 2011). Another fact that supports a divergence in splicing process between plants and other eukaryotes is the gene structure, as in general plant gene introns are shorter than mammalian introns (Reddy et al., 2013). Interestingly, plants, as shown from *Arabidopsis thaliana*, make use of AS for regulation at higher rate than animals do (Martín et al., 2021).

**FIGURE 1:**
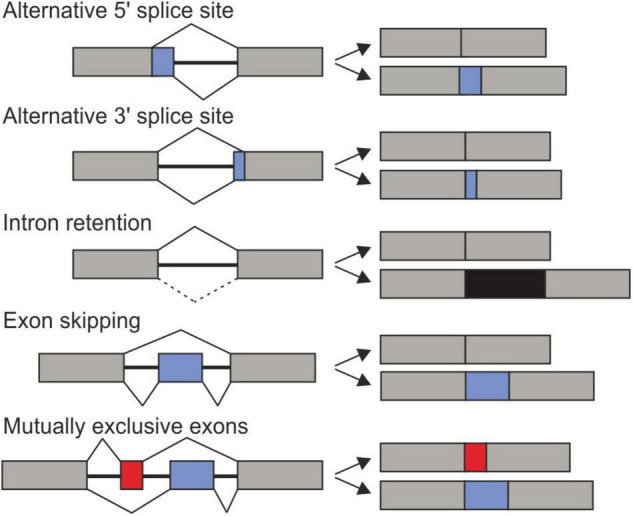
Main types of alternative splicing events. Exons are shown as boxes and black line represent introns. Constitutively expressed exons are exhibited in gray and alternatively spliced intorns are black, red or blue.

The selection of alternative splice sites allows the production of multiple mRNAs variants from a single gene. There are several modes of AS: Alternative 3′SS, alternative 5′SS, intron retention (IR), exon skipping (cassette exons), and mutually exclusive exons (Figure 1). On the one hand, many transcript variants encode for protein isoforms with distinct properties, function, localization, or stability. On the other hand, non-productive AS regulates the abundance of gene products by coupling transcription to nonsense-mediated decay (NMD) (Simpson et al., 2007; Ohtani and Wachter, 2019). Therefore, AS expands proteomic diversity and regulates gene expression at the post-transcriptional level.

Alternative splicing is regulated by the activity of *trans*-acting factors binding to *cis*-regulatory elements within the pre-mRNA (Reddy et al., 2013; Staiger and Brown, 2013). Depending on the outcome and location, these sequences are called intronic splicing enhancers (ISE), intronic splicing silencers (ISS), exonic splicing silencers (ESS) or exonic splicing enhancers (ESE) (Reddy, 2007). Binding of *trans*-acting factors to these elements can favor the usage of an otherwise suboptimal/weak splice site, change the RNA structure or cause masking of binding sites for the U1 or U2 snRNP complexes. Among the *trans*-acting factors, the families of serine/arginine-rich proteins (SR proteins), heterogeneous nuclear ribonucleoproteins (hnRNPs) and polypyrimidine tract-binding proteins (PTB) are considered as core players of AS (Lambermon et al., 2000; Lorković et al., 2000). Splicing regulators can direct the spliceosome by various ways: (a) by blocking splicing through direct competition, e.g., by interfering with snRNP binding to RNA; (b) be directing binding to snRNPs; and (c) by facilitating splicing through bridging 5′SS and 3′SS (Robberson et al., 1990).

The majority of splicing events are co-transcriptional, and thereby splicing profiles can be affected by RNA polymerase II elongation rate and chromatin structure, including histone and DNA modifications (Jabre et al., 2019). Therefore, the selection of splicing sites and generation of the final mRNA is a complex process which includes the concomitant action of many factors.

## The Global Effect of High Temperature on Alternative Splicing

Alternative splicing is considered as an important regulatory mechanism for plant survival and acclimation to stress conditions. Several studies have shown the impact of high temperatures on the splicing profiles of a major fraction of the transcriptome (Walters et al., 2013; Chang et al., 2014; Jiang et al., 2017; Keller et al., 2017; Ling et al., 2018; Lee and Adams, 2020). In the majority of studies, there is an enrichment of IR events under high temperatures. Interestingly, IR is repressed in *Physcomitrella patens* but alternative donor/acceptor site and exon skipping are induced, indicating possible differences between vascular and non-vascular plants (Chang et al., 2014). Repression of intron splicing is temperature-dependent for many genes, as increased temperatures are associated with a higher number of genes undergoing IR (Jiang et al., 2017).

Interestingly, variations in the global AS have been observed among closely related species, as in the example of *Arabidopsis thaliana* and *Boechera depauperata*, or even among cultivars of the same species such as tomato (*Solanum lycopersicum*) cultivars Moneymaker and Red Setter (Keller et al., 2017; Kannan et al., 2018). Such differences might be due to variations in *cis*-elements, or variations in the activity of splicing regulators.

Intron retention is more commonly associated with frame shifts in the coding sequence and generation of premature termination codons (PTC) (Drechsel et al., 2013). The presence of an unspliced intron and the extended 3′-untranslated region (UTR) are quality control signals for the cytosolic mRNA surveillance system NMD (Ner-Gaon et al., 2004). Consequently, IR but other AS types as well are considered to reduce transcriptome abundance and protein synthesis under proteotoxic conditions (Ling et al., 2017). This mechanism can potentially reduce the burden of the otherwise overloaded chaperone and proteasome systems under HS conditions. Worth noticing is that the more thermosensitive pollen from the tomato cultivar Red Setter shows increased IR already under non-stress conditions (Keller et al., 2017). However, in contrast to humans where genes with a pivotal role in stress response and thermotolerance such as HSFs and HSPs are more likely to undergo AS (Shalgi et al., 2014), in plants, AS seems to have a wide range of target pre-mRNAs, including abiotic stress stimuli related genes, RNA processing, transcription factors including HSFs and protein folding, including HSPs (Walters et al., 2013; Chang et al., 2014; Jiang et al., 2017; Keller et al., 2017; Ling et al., 2018; Lee and Adams, 2020). Therefore, whether AS is as beneficial as assumed based on the maintenance of proteostasis model, or detrimental due to the reduced capacity of the cell to synthesize proteins with protective functions for survival is not clear. Interestingly, pre-acclimated plants show reduced activation of AS events in response to a severe HS incident compared to non-acclimated plants (Ling et al., 2018; Sanyal et al., 2018). Regardless of which is the prevailing effect, these results highlight the importance of AS for heat stress response and thermotolerance.

## Gene-Specific Studies Showcasing the Significance of Alternative Splicing for Plant Thermotolerance

**FIGURE 2:**
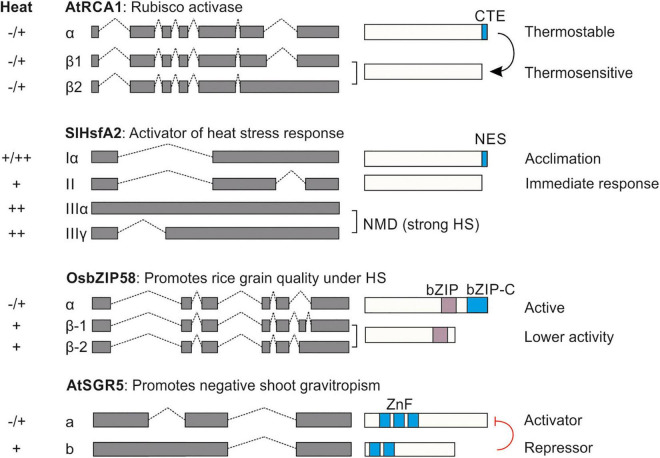
Relevance of alternative splicing for thermotolerance. Four examples for the effect of AS on the generation of protein isoforms with distinct functions. The presence of the isoform under control (–), mild (+) and strong (++) heat stress conditions is shown on the left. CTE, C-terminal extension; NES, nuclear export signal; bZIP-C, C-terminal basic leucine zipper; ZnF, Zinc finger. The black arrow in AtRCA1 indicates protection of the thermosensitive isoform. The red line indicates inhibition. Details for each case can be found in see section “Gene-Specific Studies Showcasing the Significance of Alternative Splicing for Plant Thermotolerance”.

Undoubtedly, the regulation of AS under elevated temperatures plays a significant role in the stress acclimation of plants. While the global effect of AS on the transcriptome is documented, currently the relevance of only a handful of cases have linked these events to stress response and thermotolerance (Ling et al., 2021). Here, we present four cases in which IR, A5′SS or A3′SS lead to protein isoforms with distinct functions (Figure 2).

Alternative splicing regulates the photosynthetic capacity of many plants under HS. Elevated temperatures reduce the activity of Rubisco activase (RCA) which leads to the deactivation of ribulose-1,5-bis-phosphate carboxylase/oxygenase (Rubisco) (Salvucci et al., 2006). In many plant species such as Arabidopsis, spinach and rice, AS of RCA produces multiple isoforms (Werneke et al., 1989; To et al., 1999). The long isoform (RCA-L or AtRCA1α) is more thermostable than the short as shown in *in vitro* studies, and it is hypothesized that it protects the thermolabile shorter RCA-S (or AtRCA1β1) under heat stress (Crafts-Brandner et al., 1997). In rice, only plants overexpressing the RCA-L are more thermotolerant while plants overexpressing RCA-S have an enhanced net photosynthetic rate under control conditions (Wang et al., 2010). A third shorter isoform in Arabidopsis (AtRCA1β2) which accumulates under HS shows increased stability over the other two mRNAs (DeRidder et al., 2012). Therefore, AS contributes to photosynthesis capacity in some plants by regulating the activity of RCA under high temperatures (Figure 2).

Emphasis has also been given to the regulation of HSFs by AS. All eukaryotic HSFs have a conserved intron in the DNA binding domain (DBD) coding region (Scharf et al., 2012). For many HSFs, AS in this region results in the generation of aberrant transcripts that are targeted for NMD, as shown for Arabidopsis and tomato HsfA2 (Sugio et al., 2009; Liu et al., 2013; Hu et al., 2020b). Interestingly for Arabidopsis HsfA2, under severe stress conditions, AS in this region leads to the synthesis of an mRNA that can be translated despite the long 3′UTR that classifies it as an NMD target (Liu et al., 2013). The retention of the part of the intron downstream of the miniexon creates an mRNA that codes for a truncated HsfA2 isoform that is missing a major portion of the DBD but has a hydrophobic tail, rich in phenylalanine residues. Ectopic expression of this variant in Arabidopsis plants results in an induced accumulation of endogenous HsfA2 and enhanced thermotolerance (Liu et al., 2013). The authors proposed that other HSFs, but also other TFs involved in stress response might undergo the same regulation. However, this needs to be experimentally corroborated. At least in tomato, *HsfA2-IIIα* and *HsfA2-IIIγ* produced due to full or partial retention of this intron do not produce a similar hydrophobic tail and therefore are most likely targeted for degradation (Figure 2).

Interestingly, HSFs have additional introns in some species. AS of a second intron of tomato HsfA2 leads to the generation of two protein isoforms with distinct properties: HsfA2-I is maintained in the cytosol in pre-acclimated plants due to the presence of a nuclear localization signal (NES) at the C-terminus, and is activated in case of an upcoming HS incident, contributing to ATT and probably thermomemory (Hu et al., 2020b; Figure 2). HsfA2-Iα produced by intron retention is present under a wide range of temperatures; HsfA2-II is produced only under mild HS conditions, when splicing efficiency is high. Due to the lack of an NES, HsfA2-II has a high nuclear retention, which is associated with an enhanced turnover by nuclear proteasomal degradation. Therefore, the acclimation of tomato to HS is dependent on the AS of HsfA2 (Hu et al., 2020b). Worth mentioning, tomato HsfA7 is subjected to the same regulatory control like HsfA2, further highlighting the important of AS for thermotolerance (Mesihovic et al., 2022).

In lily, LlHsfA3A-I induces LlHsfA3B under HS. In turn, AS in LlHsfA3B produces the isoform LlHsfA3B-III which interferes with the oligomerization of LlHsfA3A-I and thereby acts as a repressor of LlHsfA3-A activity through a negative feedback regulatory loop (Wu et al., 2019).

OsbZIP58 is a key factor for the regulation of storage material in rice grains during HS (Xu et al., 2020). Under HS, AS of OsbZIP58 results in two protein isoforms, the full length OsbZIP58a and the shorter one OsbZIP58b (Figure 2). The truncated isoform has lower activity. The efficiency of OsbZIP58 pre-mRNA splicing is higher in more thermotolerant rice varieties, suggesting a direct link between pre-mRNA splicing and HS seed resilience (Xu et al., 2020).

SHOOTGRAVITROPISM 5 (SGR5) is involved in the early events of gravitropic response in inflorescence stems of Arabidopsis (Morita et al., 2006). AS in SGR5 produces two protein isoforms, SGR5a and SGR5b (Kim et al., 2016; Figure 2). Under high temperatures, AS of SGR5 favors the synthesis of SGR5b, which in turn inhibits SGR5a function by forming non-functional heterodimers. By this strategy, plants can modulate negative gravitropism under high temperatures to protect the shoots from hot air (Kim et al., 2016).

## Regulation of Heat Stress Sensitive Alternative Splicing

### Role of Splicing Regulators in Heat Stress Responsive Alternative Splicing

Proteins involved in splicing are classified into protein components of the spliceosome, termed snRNPS, and spliceosome-associated proteins, termed non-snRNP proteins. Among the non-snRNP proteins, hnRNPs and SR proteins are the most extensively studied splicing regulators (Reddy, 2007). SRs and hnRNPs are modular proteins which exert their function by direct binding to intronic and exonic *cis*-elements and can therefore act as enhancers or silencers of splicing, but also by direct interactions with the core snRNPs of the spliceosome (Reddy, 2007).

Plant SR protein can interact with U1-70k and U2AF65a, the subunits of U1 and U2 snRNPs, respectively (Golovkin and Reddy, 1998, 1999; Tanabe et al., 2007, 2009; Yan et al., 2017). No report on the functional relevance of a plant SR on heat stress response and thermotolerance has been reported so far to the best of our knowledge. However, in mammalian cells, SRp38 (SRSF10) is involved in the regulation of splicing repression upon heat stress (Shin et al., 2004). Phosphorylated SRp38 is a splicing activator under control conditions. Under heat stress, the serine/threonine phosphatase PP1 dephosphorylates SRp38, leading to splicing inhibition by interaction of SRp38 with the U1 snRNP (Shin et al., 2004). The recovery of splicing is mediated by the rephosphorylation of SRp38 which is facilitated by HSP27 (Marin-Vinader et al., 2006). In mammalian cells, the CDC-Like Kinase is considered to act as thermosensor by modulating the phosphorylation of SRs under different temperatures and thereby linking changes in ambient temperatures to pre-mRNA splicing regulation (Haltenhof et al., 2020). Furthermore, hnRNP K is involved in splicing of *HSP105* pre-mRNA, highlighting the importance of both hnRNP and SR proteins for the regulation of splicing under high temperatures (Yamamoto et al., 2016).

In plants, neither a function of SRs nor of hnRNPs in heat stress sensitive AS has been shown. Nevertheless, Arabidopsis RS40, RSZ22, and SCL30 are involved in cold acclimation and acquisition of freezing tolerance (Dikaya et al., 2021). However, the induction of several SR coding genes and particularly the accumulation of protein coding transcript variants supports a putative implication of some SRs in heat stress responsive AS (Palusa et al., 2007; Rosenkranz et al., 2021). In addition, a priming heat stress treatment induces specific SR protein isoforms, which is assumed to contribute to the splicing thermomemory of plants (Ling et al., 2018; Sanyal et al., 2018). Interestingly, the induction of some tomato SR genes is dependent on the activity of HSFs, highlighting the possibility for a feedback mechanism between transcriptional and pre-mRNA splicing regulation (Rosenkranz et al., 2021).

Although the role of SRs and hnRNPs on AS under heat stress conditions is yet to be uncovered, several other proteins involved in pre-mRNA splicing have been shown to be important in this process. The splicing factor 1/branchpoint binding protein (SF1/BBP) recognizes and binds to the branchpoint sequence, and interacts with U2AF proteins, recruiting the U2 snRNP (Zhu et al., 2020). SF1 is involved in both flowering and thermotolerance by taking part in the AS of FLOWERING LOCUS M (FLM) and HsfA2 pre-mRNAs (Lee et al., 2017).

The multifunctional Sm-like snRNP proteins make the core of the U6 snRNP (Achsel et al., 1999). *A. thaliana* LSM5, along with LSM2-LSM8 rings, regulate pre-mRNA splicing (Golisz et al., 2013). An *lsm5* mutant enhances AS by promoting the inaccurate selection of splice sites and causes a higher abundance of IR transcripts. *lsm5* is thermosensitive, a phenotype that can be in part attributed to the missplicing of *AtHsfA3* but also of a DnaJ-domain chaperone protein (Cui et al., 2014; Okamoto et al., 2016).

The splicing of *AtHsfA3* and of several HSPs under heat stress is also regulated by STABILIZED1 (STA1) (Kim et al., 2017, 2018). Consequently, *sta1-1* mutants are defective in thermotolerance acquisition (Kim et al., 2017, 2018). STA1 is a plant ortholog of yeast PRP6, which is involved in the assembly of U4/U6-U5 snRNP complex by interacting with U5-snRNP and therefore has a direct role in splicing (Lee et al., 2006; Ben Chaabane et al., 2013; Dou et al., 2013).

The ski-interacting protein (SKIP) is involved in both transcription and pre-mRNA splicing (Simpson et al., 2019). The ortholog of SKIP in *Glycine max*, GAMYB, is heat stress induced and its overexpression in *A. thaliana* increases thermotolerance (Zhang et al., 2013). The knockdown of the Arabidopsis SKIP increases the thermosensitivity, highlighting the importance of SKIP for stress response, but it is not clear whether its role can be attributed to the involvement in transcription or pre-mRNA splicing.

Considering the high impact of high temperatures on AS both on qualitative (splicing type) and quantitative levels (number of genes, effect on splicing profile change), we assume that the repertoire of factors involved in heat stress sensitive AS will be enriched in the future.

### Regulation of Splicing Regulators Under Heat Stress

Alternative splicing is considered to be regulated by the abundance and post-translational modification of splicing factors. Particularly considering that with few exceptions, direct evidence for the implication of specific factors in pre-splicing under heat stress is missing, knowledge on the regulation of such factors under high temperatures can provide insights into the modulation of heat stress sensitive AS.

**FIGURE 3:**
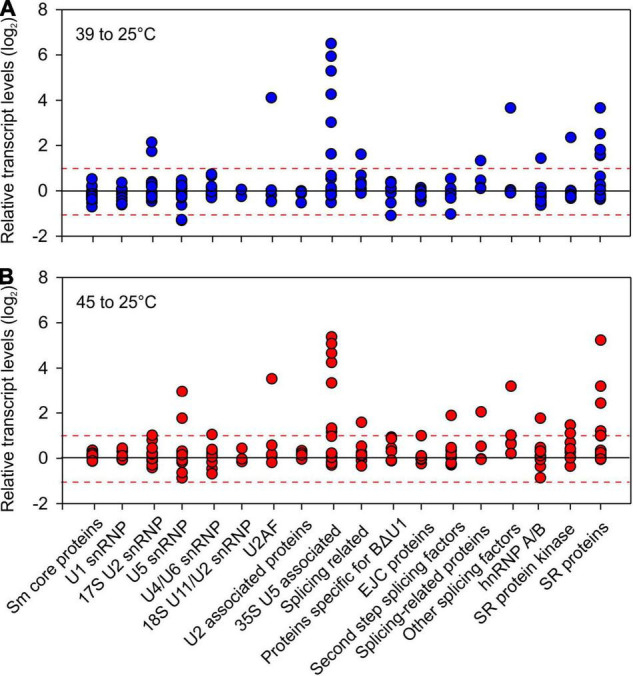
Relative transcript levels of tomato orthologs of genes involved in pre-mRNA splicing. Tomato orthologous genes of splicing related proteins were obtained by ASRG database (Wang and Brendel, 2004) using InParanoid (Sonnhammer and Östlund, 2015; Simm et al., 2016). The transcript levels were obtained from Hu et al. (2020a) on seedlings from four cultivars exposed to **(A)** 39°C or **(B)** 45°C for 1 h and are presented as relative to 25°C values.

Heat stress affects several SR genes, both on transcript and AS levels. In Arabidopsis, a comparative analysis of the splicing patterns of SR genes among different abiotic stresses showed that heat has a more dramatic effect than other stresses (Palusa et al., 2007). In tomato, five SR genes and one non-canonical SR protein, the Arabidopsis ortholog of SR45a, are induced by HS (Rosenkranz et al., 2021). Interestingly, SR subfamilies contain both stress-induced as well as non-induced members, suggesting functional diversification (Rosenkranz et al., 2021). AS in maize SR45a leads to the preferential synthesis of isoforms with greater RNA splicing potential, however, the role of SR45a in heat stress sensitive AS remains to be established (Li et al., 2021). Beyond SRs, other genes coding for pre-mRNA splicing proteins are HS-induced, both in response to mild and severe HS as shown in tomato (Figure 3 and Supplementary Table 1).

The presence of heat stress elements (HSE) in the promoters of HS-induced SRs as well as their weaker induction in an HsfA1a-knockdown transgenic line show dependency on transcriptional regulation by the HSF system (Rosenkranz et al., 2021). The presence of additional predicted *cis*-elements that can putatively be recognized by (HS-induced) transcription factors other than HSFs, as well as their only partial suppression in the HSFA1a suppression line, indicates that SRs are regulated by additional factors (Rosenkranz et al., 2021). In support of this, many SRs are induced by various abiotic stresses and hormone treatments, including ABA (Cruz et al., 2014).

Heat stress causes the accumulation of specific variants, and in some cases, the accumulation of protein coding transcripts is favored over NMD targets (Palusa and Reddy, 2010). In few cases, more than one putative SR protein isoforms have been predicted (Isshiki, 2006; Palusa et al., 2007; Chen et al., 2019b; Rosenkranz et al., 2021). Therefore, along with transcriptional regulation, AS controls the abundance of SR proteins. Furthermore, under heat stress conditions, there is a preferential recruitment of specific variants to polysomes, suggesting selective translation under high temperatures as another level of regulation (Palusa and Reddy, 2015).

Remarkably, elevated temperatures even for a relatively short time affected protein levels of many transiently expressed SRs under a CaMV 35S promoter in tomato mesophyll protoplasts (Rosenkranz et al., 2021). Some SRs showed a hypersensitivity with reduced protein levels even under mild heat stress conditions, while others were resistant and remained unaffected even under stronger HS. Interestingly, the protein stability levels of SR proteins do not correlate with their transcript profiles in response to HS (Rosenkranz et al., 2021). It can be envisioned that for some SRs, a transcriptional upregulation and/or accumulation of protein coding transcripts can at least compensate the effect of reduced protein stability.

Many splicing regulators undergo AS generating transcripts that are targeted for NMD, including SR and PTB genes (Kalyna et al., 2012). The existence of conserved alternative splice sites in many of these genes in lower and higher plants demonstrates the importance of this mechanism for gene expression of central regulators (Kalyna et al., 2006). Interestingly, some IR splice variants are NMD resilient, albeit having features assumed to be required for degradation, such as a PTC (Kalyna et al., 2012). Such intron-containing transcripts escape NMD by remaining in the nucleus and might be processed post-transcriptionally when stress has been concluded (Gohring et al., 2014).

Nuclear speckles, also termed splicing speckles or interchromatin granule clusters, have been described as sites for splicing factor storage and modification (Galganski et al., 2017). They represent a type of membraneless subnuclear organelle and reside in the interchromatin space. In both humans and plants, they are often found in close proximity to active transcription sites. They change in size and dynamics depending on transcriptional activity, likely providing splicing factors to sites of active transcription for efficient splicing of nascent transcripts (Reddy et al., 2012).

SR proteins shuttle between the nucleoplasm and speckles, whereby their nuclear localization and subnuclear distribution is determined by the phosphorylation status of their C-terminal RS domain (Ali et al., 2003; Fang et al., 2004; Tillemans et al., 2005, 2006; Rausin et al., 2010). Arabidopsis GFP-SR45 containing speckles increased in size upon prolonged heat stress, while pharmacological inhibition of transcription also had a similar effect (Ali et al., 2003). The sequestration of splicing factors such as SRs into nuclear bodies may affect splicing and in turn redirect the transcriptome landscape, for example toward the synthesis of molecules involved in the cell defense to thermal stress.

SR protein are phosphorylated in speckles which leads to their release and redirection to nascent pre-mRNAs (Ngo et al., 2005). Conformational changes caused by phosphorylation influences the interaction of the RS domain with proteins and RNA (Xiang et al., 2013). During splicing, SRs are dephosphorylated and re-enter speckles in a hypophosphorylated state (Long et al., 2019).

The phosphorylation of SR proteins in plants is mediated by Clk/LAMMER type kinases, mitogen activated kinases (MAPK) as well as SR-protein kinases (SRPK) (Savaldi-Goldstein et al., 2003). In Arabidopsis, the CDKC2 kinase accumulates in enlarged nuclear speckles which further increase in size upon heat stress treatment (Kitsios et al., 2008). Arabidopsis CDCK2 colocalizes with spliceosomal proteins such as SRs and thus is assumed to regulate components of the pre-mRNA processing apparatus (Kitsios et al., 2008). In human cells, SR proteins are dephosphorylated under HS and re-phosphorylated after stress removal. CLK1 phosphorylates SRSF9 and is required for stress body dependent IR (Shi and Manley, 2007). During recovery, the phosphorylation rate of SRSF9, but also SRSF1, is accelerated by the recruitment of CLK1, leading to IR. Interestingly, body temperature cycles drive the rhythmic phosphorylation of SR proteins as a means of AS control (Preußner et al., 2017) through a process that involves CLK kinases which are therefore considered as thermo-sensors of the circadian clock (Haltenhof et al., 2020).

Splicing factors and regulators are subjected to various post-translational modifications, including SUMOylation (Pozzi et al., 2018). SUMOylation of SR proteins has been suggested to affect both protein fate and complex assembly (Pozzi et al., 2017). SUMOylated rice OsFKBP20-1b accumulates under heat stress and the protein modification was shown to be crucial for sustaining RNA processing under high temperatures (Park et al., 2021). Interestingly, OsFKBP20-1b interacts with OsSR45 and increases its stability, thereby influencing the abundance of a major splicing regulator under stress conditions (Park et al., 2020, 2021).

### Co-transcriptional Regulation of the Heat Stress-Induced Alternative Splicing

Pre-mRNA splicing mainly occurs co-transcriptionally (Li et al., 2020), and at large is determined by RNAPII elongation, therefore the process of transcription and pre-mRNA splicing are temporally and mechanistically coupled (reviewed by Jabre et al., 2019). Consequently, factors that affect RNAPII processivity such as DNA methylation, histone chaperones and nucleosome remodelers, as well as DNA topology and histone marks contribute to pre-mRNA splicing regulation.

Currently, two models support the RNAPII and pre-mRNA splicing interplay. Elongating RNAPII with a Ser 5P CTD associates with spliceosomes, supporting a recruitment coupling model, in which (Zhu et al., 2018) the elongating RNAPII can recruit RNA processing enzymes, including splicing factors, through direct interaction with its phosphorylated carboxyl-terminal domain (CTD) repeats of its largest subunit (Bentley, 2002; Muñoz et al., 2010; Dujardin et al., 2014). In support of this model, transcription elongation factors have also been co-purified with splicing factors and spliceosomal components like U1, U2, and U5 among others (Antosz et al., 2017).

In addition to the direct interaction of RNAPII with splicing factors, the elongation rate can affect AS as well, described as kinetic coupling model (Naftelberg et al., 2015; Godoy Herz and Kornblihtt, 2019). A mutated human RNAPII with slower elongation rate favors exon inclusion compared to wild-type RNAPII *in vivo* in some genes, as a weak splice site is presented upstream of a strong splice site (De La Mata et al., 2003). The kinetic coupling model has been exemplified on the light dependent regulation of pre-mRNA splicing in Arabidopsis (Godoy Herz et al., 2019). For a set of genes, RNAPII elongation is slower in darkness but faster under light conditions (Petrillo et al., 2014) and in agreement with the kinetic coupling model, a TFIIS mutant with defected RNAPII elongation shows altered splicing profile of several genes (Dolata et al., 2015). The Arabidopsis TFIIS gene is induced during the early stages of heat stress response in an HSF-dependent manner and *tfIIs* mutants are hypersensitive to heat stress compared to wild type plants (Szádeczky-Kardoss et al., 2022). The absence of TFIIS causes changes in the AS profile of many genes under heat stress conditions, highlighting the importance of kinetic coupling with transcription for regulation of pre-mRNA under high temperatures.

Nucleosome remodeling mediates chromatin accessibility and thus, is an important integral part of heat stress response contributing to stress acclimation (Brzezinka et al., 2016). Heat stress causes a dramatic genome wide reduction in the number of nucleosomes that are associated with DNA, including heterochromatic regions (Pecinka et al., 2010). The restoration of the transcriptome landscape and the re-silencing of stress induced genes is accompanied by the reloading of nucleosomes. The regulation of gene stress responsiveness on the chromatin level requires the activity of remodeling complexes such as the SWI/SNF remodeler BMR and the associated helicase FGT1, both being essential for heat stress memory (Brzezinka et al., 2016).

Nucleosome occupancy also affects AS by mediating RNAPII elongation (Naftelberg et al., 2015). Nucleosome occupancy is higher in exons, which is assumed to contribute to the definition of exon/intron to coordinate RNAPII (Tilgner et al., 2009). The elongation speed of RNAPII is reduced in nucleosome-rich exons which is assumed to provide more time for the recruitment of splicing factors to weaker splicing sites (Muniz et al., 2021). Interestingly, the nucleosome occupancy and density also differ in constitutive and alternative exons, highlighting the role of nucleosome in AS (Wang et al., 2018).

HSF-mediated induction is dependent on the eviction of H2A.Z, a conserved variant of the canonical H2A histone, whereby the presence of H2A.Z in gene bodies restricts the transcription of heat stress induced genes under control conditions (Cortijo et al., 2017). In *Saccharomyces cerevisiae*, a relation of H2A.Z and splicing regulation is indicated by pre-mRNA splicing defects along with impairment of transcriptional regulation in cells lacking H2A.Z (Neves et al., 2017). Moreover, H2A.Z was shown to be associated with U2 snRNP-associated proteins (Neves et al., 2017). Interestingly, the depletion of Prp43, a factor that is involved in the disassembly of the spliceosome after the release of mature mRNA, leads to the suppression of the H2A.Z-mediated effects on splicing (Arenas and Abelson, 1997). Therefore, the eviction of H2A.Z in heat stress responsive genes might contribute to the AS of genes coding for essential proteins for thermotolerance, such as HSFs and HSPs.

Histone modifications are tightly related to gene expression regulation and maintenance of memory capacity through the chromatin (Lämke and Bäurle, 2017) and are associated with AS. For example, the mammalian Bromodomain protein 4 (BRD4) binds acetylated histones and can interact with pTEFb (positive transcription elongation factor b) to mediate the transition from abortive to productive elongation of RNAPII (Yang et al., 2005). BRD4 is also involved in splicing regulation, as it interacts with the JmjC domain-containing protein 6 (JMJD6), which mediates the 5-hydroxylation of U2AF65 (U2 small nuclear RNA auxiliary factor 65) (Webby et al., 2009). Consequently, BRD4-depleted cells showed increased splicing inhibition, while *Saccharomyces cerevisiae* mutants of the ortholog BDF1 exhibit a reduced recruitment of U1 snRNP in intron containing genes (Albulescu et al., 2012). Interestingly, knockdown of human BRD4 caused increased IR under HS, suggesting that BRD4 prevents cells from heat stress-induced splicing inhibition (Hussong et al., 2017).

Another example is the change in splicing profiles of genes related to flowering such as FLOWERING LOCUS M (FLM) in mutants of the H3K36 methyltransferases *SET DOMAIN-CONTAINING GROUP 8* (*SDG8*) and *SDG26* in Arabidopsis (Pajoro et al., 2017). While this has been shown for mild temperature increases, it is not known whether this hold true for conditions that cause heat stress. Yet another example is the Arabidopsis PROTEIN ARGININE METHYL TRANSFERASE 5 (PRMT5) that methylates arginine residues in histones and Sm spliceosomal proteins, with the latter being related to the modulation of 5′-splice-site recognition, thereby linking the circadian clock to the regulation of AS in plants (Sanchez et al., 2010).

Not only modification of histones, but also modification of the DNA itself has been suggested to influence AS. DNA methylation, the result of the addition of a methyl group on cytosine bases to form 5-methylcytosine, is an important epigenetic modification in plants. Since differential DNA methylation patterns are strongly correlated with nucleosome occupancy, they may influence RNAPII elongation speed and splicing factor recruitment, resulting in alterations of splicing profiles (Maor et al., 2015). CG methylation is more abundant in exons than in introns, and mutation of the CG methyltransferase *OsMet1-2* resulted in altered splicing profiles of several genes, providing evidence for a link between DNA methylation and pre-mRNA splicing (Yamauchi et al., 2008). This is further supported by differences in the methylation levels between constitutive and alternative introns in cotton (Wang et al., 2018).

In mammals, three factors have been identified so far to directly link DNA methylation and pre-mRNA splicing (Maor et al., 2015). In mammalian cells, the CCCTC-binding factor (CTCF) binds unmethylated DNA to reduce the RNAPII velocity and thereby regulate splicing through kinetic coupling (Shukla et al., 2011). The methyl-CpG binding protein 2 (MeCP2) has the opposite function; it recruits histone deacetylase (HDAC) to stimulate histone hypoacetylation and thereby reduce RNAPII elongation (Young et al., 2005). Thereby, DNA methylation in exons causes exon skipping through activity of MeCP2 (Maunakea et al., 2013). While CTCF and MeCP2 affect splicing via modulating the speed of RNAPII, Drosophila heterochromatin protein 1 (HP1) binds to methylated DNA and recruits splicing factors onto the pre-mRNA, including SRs and hnRNPs (Loomis et al., 2009; Piacentini et al., 2009). Although plants do not possess CTCF, a similar effect of other methylation-dependent DNA-binding factors on either RNAPII activity or splicing factor recruitment cannot be excluded (Dong et al., 2020). Supporting this notion, DNA methylation at splice sites, as for example CHG in splice acceptor sites, has been proposed to contribute to splicing efficiency and/or AS in a process that might involve small RNA and histone K3K9 methylation (Regulski et al., 2013).

### Regulation of Heat Stress Sensitive Splicing by Pre-mRNA Structure

Alternative splicing is mediated by the binding of regulatory proteins to motifs acting as silencers or enhancers. Furthermore, the secondary structure of the pre-mRNA can influence the binding of these proteins and therefore affect the splicing outcome (Hiller et al., 2007; Shepard and Hertel, 2008; Liu et al., 2021). Regulatory proteins preferentially bind single-stranded RNA, and therefore changes that cause the formation of double strands might prevent their binding (Auweter et al., 2006). For example, the formation of loop structures out of the alternative spliced exons has been proposed to prevent exon recognition (Miriami et al., 2003; Zhang et al., 2005).

In prokaryotes, the role of changes of RNA structure in response to temperature is a major regulatory mechanism for translation control, typically by blocking the binding of the ribosome under control temperatures, and then exposing them under high temperatures via melting of the RNA structure (Winkler and Breaker, 2005). Such RNA thermometers are present in PIF7 and HsfA2 in Arabidopsis and contribute to translation of these two genes under aberrant temperatures (Chung et al., 2020). Heat stress causes an intensive reprogramming of the RNA structurome in rice and although not investigated so far, such changes could affect the splicing profile of several genes (Su et al., 2018). In yeast, the presence of an RNA fold in the 3′ splice site modulates AS under heat stress conditions, thereby acting as a thermosensor (Meyer et al., 2011).

Differences in the splicing pattern of HsfA2 between haplotypes from modern cultivars and wild tomato species have been attributed to three intronic singly nucleotide polymorphisms (SNPs) (Hu et al., 2020b). Based on nuclear magnetic resonance (NMR) analysis, the presence of SNPs alter the local RNA structure and thus might affect the binding of splicing regulators (Broft et al., 2022). Interestingly, while increased temperature did not affect the structure of the 5′-end of the intron, a 3′-end intronic region shows a temperature dependent RNA fold, yielding a highly stable structure under high temperature (Broft et al., 2022). It is hypothesized, that the stable structure unfolds a single stranded region that might facilitate the binding of splicing silencers.

## Methods for the Characterization of Alternatively Spliced Rnaome Under Heat Stress

Understanding the effect of high temperatures on alternative splicing requires a qualitative and quantitative description of the plant RNAome. Here, we provide a list of genomic techniques that have or can be used to get information about AS.

High throughput sequencing is a fast and affordable technique to determine the whole RNAome expression levels (De Paoli-Iseppi et al., 2021). Second generation sequencing technologies like Illumina or Ion Torrent produce huge amounts of an average 100–200 nt long reads leading to high coverage with a trade-off for accuracy for isoform characterization and identification (Trapnell et al., 2012; Engström et al., 2013). A main drawback of this approach is the determination of the combination of short reads to characterize isoforms as they are hard to map unambiguously. Typically, the splice-aware components are based on exon-first (unspliced mapping of reads and later detection of connections between read cluster) or seed-and-extend (k-mer/substring mapping and extending until threshold) approaches (Alamancos et al., 2014). Nowadays, AS analysis is performed preferentially with long-read sequencing technologies (Oikonomopoulos et al., 2020). These so-called 3rd generation sequencing methods by Pacific Biosciences (PacBio) and Oxford Nanopore Technologies (ONT) renounce a high coverage in favor of the creation of up to 10 kb long-reads covering the entire isoform. Further, the different sequencing protocols like single-pass reads (PacBio), circular consensus sequencing (PacBio) or Oxford Nanopore have an error rate up to ∼15% (Uapinyoying et al., 2020). By this, the challenging reconstruction task of isoforms is omitted but the requirement of large numbers of reads to deeply profile the RNAome is higher than the throughput that is currently obtainable (Chen et al., 2019a). Further, specific 3nd generation sequencing mappers have been invented, due to the complexity of the datasets consisting of variable length of transcripts, multiple alternatively spliced isoforms for many genes and high sequence similarity of highly abundant species of RNA (Oikonomopoulos et al., 2020). Particularly the combination of 2nd and 3rd sequencing generation platforms can be beneficial for the quantification of splice variants and characterization of the RNAome of species irrespective of the reference genome’s annotation status.

Validation of the results of the global analysis can be done for specific genes based on standard techniques such as PCR and RT-PCR which allow high sensitive detection and quantification of mRNA splice variants for single genes. Particularly, high regulation- (HR) RT-PCR which is based on the separation of the fluorescently labeled amplicons generated by RT-PCR in a capillary sequence system, allows the accurate quantification of variants even with a single base pair difference (Simpson et al., 2008).

Future attempts should deduce a detailed heat stress RNAome on a single cell level, as single-cell RNAseq (scRNAseq) can provide a more detailed picture, particularly considering that stress response and tolerance vary among different cell types (Ntranos et al., 2019). Here, the combination of long read technologies and scRNAseq will be essential to increase accuracy in isoform detection and reduce noise due to low sequencing depth and high technical variability (Liu et al., 2017).

In addition, understanding the basis of the regulation of the heat stress sensitive alternative splicing, it is important to identify *trans*-acting factors and decipher their activity on the RNAome (Köster and Meyer, 2018). Techniques that allow the immunoprecipitation of RNAs associated or bound under native or non-native conditions are an indispensable tool toward this direction. Protein-RNA interactions methods like RNA immunoprecipitation (RIP)-seq, cross-linking immunoprecipitation (CLIP)-seq and CLIP-based techniques can provide information on the association of splicing factors with specific pre-mRNAs and their binding sites (Meyer et al., 2017; Hannigan et al., 2018). The two techniques can be complementary, and their combination can provide a more deep information on the relation of splicing factors with pre-mRNA (Hafner et al., 2021).

As the activity of the splicing regulators can be dependent on the structure of the RNA, an in depth and global determination of the changes posed by high temperatures on RNA folding can provide valuable information. Recently, *in vivo* approaches such as DMS-Seq which is based on dimethyl sulfate methylation of unprotected cytosines and adenines (Ding et al., 2014; Rouskin et al., 2014) have provided insights into RNA folding and could allow correlation of RNA fold alterations with splicing profile changes. The structure of nuclear RNA of Arabidopsis has been also investigated by *in vivo* SHAPE (Selective 2′ Hydroxyl Acylation analyzed by Primer Extension)-Structure-Seq which overcomes the limitation of DMS-based techniques to solely analyze the base-pairing status of adenine and cytosine (Liu et al., 2021).

In summary, the RNAome can be analyzed under many different aspects leading to a huge variety of high throughput datasets in relation to AS. The scientific premise is to understand and predict *in silico* the “splicing code” using a combination of genomic and RNA features (Park et al., 2018). For such approaches machine learning (ML) can be used for pattern recognition, classification and prediction (Libbrecht and Noble, 2015). Deep learning approaches using neural net (NN) architectures can be used elucidate the genetic determinants based on AS variants on the basis of different stress regimes, even in a tissue or cell-type dependent manner (Xiong et al., 2015). Particularly, the combination of RNAome expression and AS information with interaction data like ChIP-seq or CLIP-seq could be used for more accurate AS prediction at least in relation to splicing factors and regulators.

## A Hypothetical Model for the Heat Stress Induced Intron Retention

**FIGURE 4:**
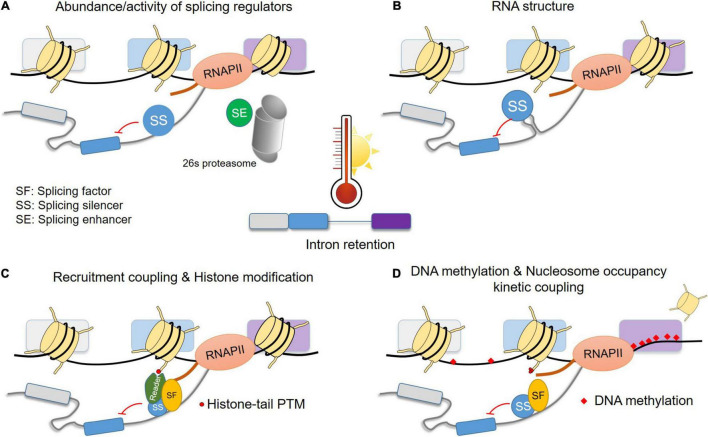
Hypothetical model for the basis of intron retention under heat stress conditions. The model is depicted for IR but can apply for A5′SS and A3′SS as well. Possible effects of high temperatures on pre-mRNA splicing by **(A)** changes in the abundance of splicing regulators, **(B)** changes in RNA structure that can influence binding of splicing regulators, **(C)** recruitment of splicing regulators by histone modifications or direct interaction with RNAPII C-terminal domain, **(D)** heat stress induced changes in DNA methylation and nucleosome occupancy which can affect the RNAPII velocity and consequently binding of splicing regulators. Only few cases are depicted and more details can be found in see section “A Hypothetical Model for the Heat Stress Induced Intron Retention” in the main text. PTM: post-translational modification. Other abbreviations are explained in the figure.

High temperatures cause an increase in AS, with partial (A3′SS or A5′SS) or complete IR to be the most prominent events. As the basis of this phenomenon is not well understood in plants, we provide hypotheses based on knowledge from different eukaryotic systems, as described above. Heat can affect the abundance and/or activity of splicing regulators. The abundance can be mediated on the transcriptional level (e.g., induction of SR coding genes), AS (e.g., synthesis of protein isoforms with competing functions or aberrant mRNAs targeted for NMD), or protein stability (e.g., degradation by 26S proteasome) (Figure 4A). In addition, post-translational modifications (e.g., phosphorylation) of splicing regulators under high temperatures can affect the activity, subcellular localization and interaction with other factors. The binding of splicing factors can be enhanced or restricted by changes in the structure of the pre-mRNA (Figure 4B). Furthermore, in co-transcriptional splicing, the recruitment of splicing factors can be mediated via reader proteins of specific histone modifications (Figure 4C). Last, under heat stress, changes in DNA methylation and nucleosome occupancy are expected to affect RNAPII velocity to some degree and consequently alter the splicing profile of genes. For example, DNA methylation in splicing sites can cause weaker splicing, while binding of proteins to methylated DNA in gene bodies can affect RNAPII velocity (Figure 4D). In some loci, a lower nucleosome occupancy might allow faster RNAPII elongation and therefore prevent intron splicing due to reduced time for the assembly of the spliceosome on the pre-mRNA. The elucidation of these mechanisms on gene and genomic levels are essential for understanding of the heat stress response and thermotolerance. As differences in thermotolerance among plant species and even among cultivars have already been attributed to variations in alternative splicing, it can be envisioned that the identification of key players of heat stress sensitive alternative splicing will support crop improvement.

## Author Contributions

SF conceived the structure of the manuscript. SF wrote the main part of the manuscript with the help of RR, SU, KL, and SS. All authors have read and approved the manuscript.

## Conflict of Interest

The authors declare that the research was conducted in the absence of any commercial or financial relationships that could be construed as a potential conflict of interest.

## Publisher’s Note

All claims expressed in this article are solely those of the authors and do not necessarily represent those of their affiliated organizations, or those of the publisher, the editors and the reviewers. Any product that may be evaluated in this article, or claim that may be made by its manufacturer, is not guaranteed or endorsed by the publisher.

## Supplementary Material

Supplementary Table 1 List of tomato pre-mRNA splicing genes and their transcript levels under different temperatures. Tomato orthologous genes of splicing related proteins were obtained by ASRG database (Wang and Brendel, 2004) using InParanoid (Sonnhammer and Östlund, 2015; Simm et al., 2016). The transcript levels were obtained from Hu et al. (2020a) on seedlings of four different cultivars kept under 25, 39 or 45°C for 1 h. Transcript levels are based on Massive Analysis of cDNA Ends (MACE) and presented as transcripts per million (TPM).
